# Population Pharmacokinetics of Valproic Acid in Pediatric and Adult Caucasian Patients

**DOI:** 10.3390/pharmaceutics14040811

**Published:** 2022-04-07

**Authors:** Paulo Teixeira-da-Silva, Jonás Samuel Pérez-Blanco, Dolores Santos-Buelga, María José Otero, María José García

**Affiliations:** 1 Pharmaceutical Sciences Department, Universidad de Salamanca, 37007 Salamanca, Spain; sbuelga@usal.es (D.S.-B.); mjgarcia@usal.es (M.J.G.); 2 Institute of Biomedical Research of Salamanca (IBSAL), 37007 Salamanca, Spain; mjotero@saludcastillayleon.es; 3 Pharmacy Service, University Hospital of Salamanca, 37007 Salamanca, Spain

**Keywords:** drug interactions, therapeutic drug monitoring, epilepsy, NONMEM, population pharmacokinetics, valproic acid

## Abstract

(1) Background: The aim of this study was to explore the valproic acid (VPA) pharmacokinetic characteristics in a large population of pediatric and adult Caucasian patients and to establish a robust population pharmacokinetic (PopPK) model. (2) Methods: A total of 2527 serum VPA samples collected from 1204 patients included in a therapeutic drug monitoring program were retrospectively analyzed. Patients were randomly assigned to either a model development group or an external evaluation group. PopPK analysis was performed on 1751 samples from 776 patients with NONMEM using a nonlinear mixed-effect modelling approach. The influence of demographic, anthropometric, treatment and comedication variables on the apparent clearance (CL/F) of VPA was studied. The bootstrap method was used to evaluate the final model internally. External evaluation was carried out using 776 VPA serum samples from 368 patients. (3) Results: A one-compartment model with first-order absorption and elimination successfully described the data. The final model included total body weight, age and comedication with phenytoin, phenobarbital and carbamazepine with a significant impact on VPA elimination. Internal and external evaluations demonstrated the good predictability of the model. (4) Conclusions: A PopPK model of VPA in Caucasian patients was successfully established, which will be helpful for model-informed precision dosing approaches in clinical patient care.

## 1. Introduction

Valproic acid (VPA) is an antiepileptic drug (AED) that has been widely used in multiple seizure types and various neurological and psychiatric disorders since its serendipitous discovery in 1962 [1]. It is still considered a first-line option for treating generalized epilepsies [2].

There are several oral formulations on the market, differing in their rate of absorption. However, regardless of the formulation, VPA absorption is rapid and almost complete with a bioavailability greater than 90% [1]. The metabolism of VPA is mainly characterized by three routes: glucuronidation via uridine diphosphate glucuronosyltransferase (UGT) isoforms (50%), including UGT1A3/1A4/1A6/1A8/1A9/1A10/2B7; beta oxidation in mitochondria (40%); and cytochrome P450 (CYP)-mediated oxidation (10%), such as CYP2A6/2B6/2C9/2C19 [3,4,5,6]. Only a small amount of VPA is excreted unaltered in urine (1–3%). VPA serum apparent clearance (CL/F) varies from 6 to 10 mL/h/kg [6], with a half-life ranging from 12 to 16 h and from 8.6 to 12.3 h for adults and children, respectively [5].

Serum level monitoring was introduced as a result of the large interindividual variability (IIV) observed in the pharmacokinetic (PK) behavior of VPA, in order to individualize dosage regimens and to achieve a steady-state serum concentration between 50 and 100 mg/L [5]. It is essential to establish adequate and robust population PK (PopPK) models of VPA and to investigate the influence of potential covariates on its PK behavior, specially to quantify drug–drug interactions with other AEDs and the PK changes from childhood to adulthood. Traditional pharmacokinetic methods make it difficult to estimate pharmacokinetic parameters from clinically obtained sparse blood samples.

VPA exhibits saturable binding to serum proteins, which results in a higher unbound fraction at high serum concentrations [7]. Some studies included VPA dose with its influence on its CL/F, probably to capture this phenomenon [7,8,9,10,11,12,13,14,15,16]. However, this nonlinear PK behavior remains controversial [7], being highlighted as a potential confounding factor of the commonly known “Therapeutic Drug Monitoring (TDM) effect”. In fact, in clinical setting, subjects with higher CL/F will have a lower concentration of the drug, and consequently higher doses will be administered and vice versa. That is why VPA dose is not recommended for consideration as a potential covariate in PopPK models based on sparse data from TDM [17].

Previous PopPK studies showed that total body weight (TBW), age, gender, genetic factors, VPA dose and comedication had a significant influence on VPA PK parameters [6,7,8,9,10,11,13,14,15,17,18,19,20,21,22,23,24,25,26,27]. However, few PopPK studies have been performed with a wide range of ages of Caucasian patients. This study was performed with the aim of exploring the PK characteristics of VPA in a large population of pediatric and adult Caucasian patients and developing a robust VPA PopPK model for improving current VPA therapeutic drug monitoring (TDM).

## 2. Materials and Methods

### 2.1. Study Design and Population Characteristics

Data from ambulatory patients (aged 0.11–92.9 years old) treated with VPA and followed by the TDM program of the University Hospital of Salamanca were retrospectively recruited for model development and external evaluation. Only mono- or dual therapies were considered, and the following exclusion criteria were applied: (1) missing laboratory data/treatment information/concentration data; (2) inaccurate medication or blood collection time records; (3) poor patient treatment adherence; (4) patients whose body mass index (BMI) was outside the range of 16.0–39.9 kg/m^2^, in accordance with World Health Organization (WHO) indications (for patients under 18 years of age, the criterion used was three standard deviations from the means in the WHO tables for children) [28]; (5) nonsteady state achieved and (6) more than two AEDs administered concurrently.

Steady-state concentrations were assumed to have been reached a month after the initiation of treatment or a dose change. Treatment adherence was assessed by means of an interview with the attending health care provider. Demographic and anthropometric information (age, gender, height (HGT), TBW, body surface area (BSA), BMI,), disease information (seizure type and diagnosis), medication information (dosage forms, dosage regimens and administration time), comedication with another antiepileptic drug other than VPA (carbamazepine (CBZ), phenytoin (PHT), phenobarbital (PB), ethosuximide (ESM), lamotrigine (LTG), topiramate (TPM) and clobazam (CLB)) and the analytical technique used to determine serum concentrations were recorded for each patient.

All patients included in the study were randomly assigned to either a model development or a model external evaluation group in an approximate 2:1 proportion stratified by age groups (Table S1). The model development dataset consisted of 1751 serum concentration samples from 836 patients. In addition, 776 serum samples from 368 different patients were used as an external evaluation dataset. Table 1 shows a summary of the baseline characteristics of both the development and external evaluation datasets. No statistically significant differences (*p* < 0.001) were shown for any covariate considered across the two datasets considered.

The final PopPK model was evaluated internally and externally using the development and external evaluation datasets, respectively, and for the last step, the two datasets (development and external evaluation) were merged for evaluating potential differences in the final PK parameter estimates. Thus, three types of datasets were used: the development, the external evaluation and the merged datasets. The effect of demographic, anthropometric and comedication variables on VPA CL/F was investigated.

### 2.2. Blood Sampling and Assay

VPA was administered orally in one of the following presentations: gastro-resistant tablets (200 mg and 500 mg), coated prolonged-release tablets (300 mg and 500 mg) or oral solution (200 mg/mL). The dose was adjusted in accordance with the observed VPA serum level, clinical efficacy and any adverse reactions. All of the blood samples were taken as a part of the routine TDM procedure. Fluorescent polarization immunoassay (FPIA) was used to determine serum VPA concentrations, using a fluorescence polarization analyzer (Abbott TDx analyzer) with an inter- and intra-assay variation coefficient of less than 10% and a limit of detection of 0.7 mg/L [31,32]. Normally, serum VPA concentrations were obtained at the end of the dosing interval and once the steady state had been reached.

### 2.3. Population Pharmacokinetic Modeling

NONMEM (v.7.5.1, ICON Development Solutions, Ellicott City, MD, USA), Perl-speaks-NONMEM (PsN) v.5.2.6. (Uppsala University, Sweden, http://psn.sourceforge.net), R v.4.1.2., RStudio v.2022.02.0+443 (RStudio, Boston, MA, USA) and Pirana v.3.0.0. (Certara, Princeton, NJ, USA, http://www.certara.com) were used to apply a nonlinear mixed-effect modeling methodology. The first-order conditional estimation method with interaction (FOCEI) was used. The log-transforming both sides (LTBS) approach was applied to the VPA concentrations [33]. Most concentrations (97.5%) in this study were steady-state trough concentrations ($C_{min}^{ss}$), which could not totally reflect the absorption and distribution process characteristics. Considering the type of sampling points ($C_{min}^{ss}$ and sparse data) and information available in the literature, a one-compartment structural kinetic model with first-order absorption and elimination was chosen as the base structural PK model [8,9,10,14,15,17,24], and the absorption rate constant (Ka) was fixed at 2.64 h^−1^ for oral solution (syrup), 0.78 h^−1^ for gastro-resistant tablets and 0.38 h^−1^ for modified-release coated tablets based on previous information [7,8]. In the case formulation information in the clinical records was not available, oral solution and gastro-resistant tablets were considered for patients younger and older than 12 years old, respectively. PK parameters scaled to TBW were a priori included in the base model based on physiological reasons and previous knowledge [34]. A standard allometric scale based on TBW with a single exponential value of 0.75 was assumed for apparent clearance (CL/F), and a single exponential value of 1.0 was defined for apparent volume of distribution (V/F). Estimation of these exponents was also evaluated. Additional maturation functions for characterizing physiological changes in addition to those explained by body size were also investigated. Furthermore, the population value of V/F was fixed at 14 L for a typical patient of 70 kg in accordance with previous studies [5]. Although these assumptions could be considered a limitation, they enable the use of data generated during clinical practice (sparse data), and this constitutes the foundations of the utility of the PopPK approach developed by Beal and Sheiner in 1990 [35,36,37].

PK parameters were assumed log-normally distributed; thus, an exponential model was used to describe the interindividual variability (IIV) in CL/F. The residual unknown variability (RUV) was included as an additive error model after the natural logarithm transformation of measured drug concentrations and model predictions (which is equivalent as a proportional error in the natural scale) [33]. Additional error models, such as additive and combined, were also tested.

Plausible variables previously identified in the literature and with physiological significance were incorporated into the model for covariate screening and identification, by means of a stepwise strategy performed in PsN (*p*-forward = 0.05, *p*-backward = 0.01). In the forward step, the selected covariates were included in the base model one by one, and only those causing a decrease in the objective function value (OFV) > 3.84 (*p* = 0.05, χ^2^ distribution with one degree of freedom) were incorporated into the full regression model. Covariates were removed one at a time in the backward elimination step performed on the final forward step model. A covariate resulting in an increased OFV of over 6.63 (*p* = 0.01, χ^2^ distribution with one degree of freedom) was considered significant for CL/F prediction and was retained in the final PopPK model. All other covariates that did not meet this criterion were excluded. Linear and exponential functions were used to analyze continuous covariates, whereas the dichotomous categorical covariates were analyzed by estimating the change in the PK parameter with respect to the reference group (most common). Finally, a conditional function was used for categorical covariates with more than two groups [38].

### 2.4. Model Assessment and Evaluation

Model selection was guided by run convergence minimization with at least 2 significant digits in parameter estimates, a successful covariance step, changes in minimum objective function value (MOFV) for each nested model (*p* < 0.05, χ^2^-test, and ΔOFV > 3.84), plausibility and precision of parameter estimates, evaluation of random effects (i.e., η and ε) shrinkage, reduction in IIV and/or RUV and visual inspection of standard diagnostic plots including goodness-of-fit plots (GOF). Performance of the final PopPK model was assessed by both internal and external evaluation. A bootstrap resampling technique from the development dataset was used to judge the reliability and stability of the final PopPK model developed in the study. A total of 1000 bootstrap-resampled datasets were generated from the original model development dataset, and each was individually fitted to the final PopPK model with PsN. All PK parameters were estimated in the 1000 bootstrap datasets, and the median and 95% confidence intervals (CI) of the parameters were compared with the estimates of the final PopPK model parameters.

The external evaluation (external evaluation dataset) of the model using different patients than those used for model development from a real (not simulated) population with similar characteristics to the population used for model development was carried out in three steps [39,40]:

(1) Evaluation of the predictive capacity of the final PopPK model developed in the external evaluation dataset, using the option MAXEVAL = 0 (Bayesian forecasting). The mean prediction error (MPE) and root mean squared prediction error (RMSE) were calculated to determine bias and precision, respectively [41]. These metrics were evaluated by age group to confirm the accurate and precise-model-based VPA predictions across the wide range of ages considered in this study.
(2) PK parameters’ re-estimation with the merged dataset (model development together with external evaluation datasets) to confirm the stability of the final PK parameter estimates when different patients were considered.
(3) Visual inspection of the GOF generated when VPAs of external evaluation dataset are predicted through Bayesian forecasting (MAXEVAL = 0) based on a priori information relying on the final PopPK model developed.

## 3. Results

### 3.1. Population Pharmacokinetic Modeling

A one-compartment model was selected as the structural model to describe VPA PK profile. Age significantly influences CL/F, which was mainly captured by the inclusion of TBW in CL/F following allometric scaling principles (IIV of CL/F decreased by 41% when only considering TBW on CL/F).

Exponential and proportional error models for IIV and for RUV, respectively, successfully described the data (Table 2).

**Figure 1:**
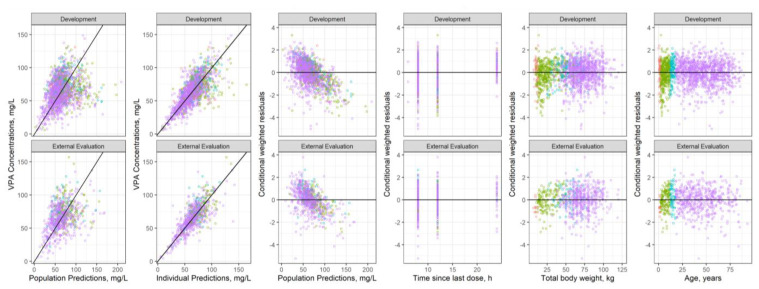
Goodness-of-fit plots for the development dataset (upper panels) and the external evaluation dataset (lower panels) colored by age classification (AGEC), ● 28 d–2 y ● 2–11 y ● 12–18 y ● >18 y.

A visual inspection of the GOF plots allowed us to confirm an adequate description of the data except for subjects weighting less than 24 kg and/or younger than approximately 6 years old (data not shown). Therefore, additional evaluations of CL/F were carried out. Thus, the potential influence of age on VPA CL/F, were evaluated both as a standard maturation function collapsing at 2 years old (Hill equation) and as an exponential relationship centered on the age of 15, the cut-off point observed following a visual inspection of CL/F with respect to age. Finally, age was included on VPA CL/F following an exponential relationship as described in Equation (1), which, together with the comedications influence on CL/F identified in the covariate model evaluation procedure, considerably improved the GOF in the youngest pediatric subjects (Figure 1).

The covariates fulfilling the statistical requirement for inclusion (*p* < 0.001) were, in addition to TBW as an allometric relationship: AGE centered on the age of 15 and introduced as a power function on CL/F and comedication with carbamazepine (CBZ), phenytoin (PHT) and phenobarbital (PB) (Equation (1)). Furthermore, the association with LTG and gender initially showed a statistically significant impact on CL/F (*p* < 0.005). However, these covariates were not retained in the final model due to the lack of clinical relevance, as the VPA CL/F was impacted by less than 10% in both cases. VPA V/F was finally described following an allometric scaling relationship with a value of 14 L for a typical adult patient (Equation (2)), as explained above.

CL/F [L/h] = 0.646 (TBW/70)^0.75^ × 1.640^PHT^ × 1.386^PB^ × 1.521^CBZ^ × (AGE/15)^−0.0154^ (1)

V/F [L] = 14 (TBW/70)^1^ (2)

where TBW is the total body weight in kg, Age is the age in years and PHT, PB and CBZ represent comedications of phenytoin, phenobarbital and carbamazepine, respectively; these variables (comedication) take a value of 0 when absent and 1 when the drug is administered simultaneously with VPA.

These results highlight the requirement of VPA dose intensification in combination with other AEDs, especially in adults and very young children (1 year old), where VPA serum concentration can be reduced by approximately 50% compared to the VPA administered in monotherapy.

The estimated CL/F of VPA for a typical patient with a median TBW of 70 kg and absence of any comedication was 0.646 L/h. Compared to the corresponding base model, the IIV of CL/F decreased by about 48%, showing that the occurrence of these covariates effectively improves the fit of the data.

Given the sampling strategy routinely applied in TDM procedures (mainly $\;C_{min}^{ss}$), V/F was not able to be suitably estimated with sufficient physiological plausibility. Thus, V/F was fixed at 14 L for a standard adult patient of 70 kg [5].

### 3.2. Model Evaluation

The bootstrap results (Table 2) demonstrate the robustness of the PopPK model. The 95% confidence interval (CI) of parameter estimates showed satisfactory overlap. The final model proved to be highly stable, with a total of 99.6% bootstrap runs fitting successfully without problems to the new datasets generated by bootstrapping. The bootstrap estimates resembled those of the population with errors lower than 50% in all the parameters, except for the RUV value, potentially due to the sampling design.

All of the PK parameter estimates with the merged dataset (including both the development and the external datasets) were within the 95% CI of the PK parameters obtained in the bootstrap analysis (Table 2) (except the RUV, as previously mentioned), demonstrating the adequate predictive power of the proposed final model (Source code of the final model is available in Table S2).

The predictive performance of PopPK was assessed by means of a comparison between the concentrations observed in the external evaluation dataset and those predicted by the final model developed. Figure S1 shows an adequate distribution of the prediction errors calculated (mostly distributed within ±30%) in the external evaluation dataset through Bayesian estimation across all the different age classification groups supporting the correct model predictability along ages. In addition, Table 3 shows the calculated MPE and RMSE for population-predicted concentrations (PRED) and individual-predicted concentrations (IPRED). Estimation errors were acceptable in the PK parameters estimated for fixed (<20%) and random effects (<6%), as well as shrinkage values (<20%).

In the case of the PK parameter quantifying the impact of the age on VPA CL/F, the standard error was high, which may limit its validity. However, bootstrap results confirm the value estimated in a largest dataset, supporting, together with the GOF, its inclusion in the final model. Internal evaluation by bootstrapping methodology demonstrated the good predictability of the final PopPK model developed (Table 3).

**Figure 2:**
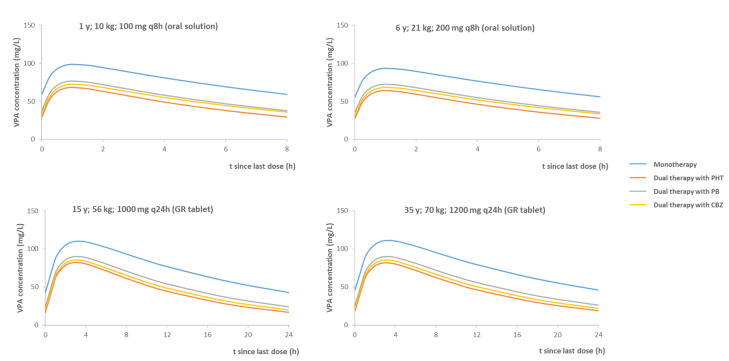
Valproic acid (VPA) concentration–time profiles simulated with the final model developed. The following acronyms represent the drug administered in each scenario: VPA monotherapy together with carbamazepine (CBZ), phenobarbital (PB) and phenytoin (PHT); GR: gastro-resistant.

Figure 2 shows the evolution of steady-state plasma VPA concentrations in different scenarios: four age groups in mono- and dual therapy, with the antiepileptic drugs identified in the PopPK. The regimens shown were designed to achieve VPA concentrations within the acceptable therapeutic margins in monotherapy.

## 4. Discussion

Individual differences in drug disposition could cause effective epilepsy management to fail. Traditionally, VPA TDM was routinely carried out in a large number of hospitals, which made it possible to individualize the dosage of this drug. To address the PK-guided TDM of VPA, robust and sufficiently evaluated and wide applicability (age and weight ranges), PopPK models are required to help optimize VPA dosage regimens. Soreducing health care costs and improving treatment outcomes by using Bayesian algorithms and drug serum concentrations taken during routine clinical monitoring.

A number of studies have developed VPA PopPK models in epileptic patients [1,3,7,8,9,11,13,14,15,16,17,18,19,20,21,22,23,24,25,26,27,42,43,44]. Most of the previous PopPK models available were developed based on non-Caucasian populations, and some of them involved a small number of patients, which limits their usefulness in the clinical setting. Furthermore, one-third of the published PopPK models for VPA were not adequately evaluated at all, and several of the remaining PopPK models did not have an external evaluation performed [6], which is highly recommended by the European Medicines Agency (EMA) and the Food and Drug Administration (FDA) [45,46], especially with studies carried out with real-world patients.

The development of this PopPK model is justified by the need for a model that has been validated both internally and externally, making it possible to correctly estimate CL/F in both children and adults, and does not include DDV as a covariate, so that it may be used in dosage optimization.

Considering the large number of data available for model development and the wide range of ages included (0.1–92.9 years old), special caution was taken to characterize PK changes according to body size and years of life related to physiological maturation. In conclusion, the impact of total body weight on VPA CL/F following an allometric relationship together with age for correcting maturation processes, mainly in young infants, adequately described the large number of data considering the broad age spectrum (Figure 1). Gender showed a slight influence on CL/F. However, this difference was not considered clinically relevant (<10% change of VPA CL/F), and gender was not retained in the final model. This finding is in agreement with other studies that tested the gender covariate in the CL/F [1,3,7,8,9,11,13,14,18,21,23,26,42,44]. However, a few studies showed that gender had a significant effect on CL/F, reporting a reduction from 5.7% to 35.4% in women [10,16,19,24,27,43].

The most important factors affecting VPA PK are those related to their association with drugs that induce or inhibit its metabolism. Consequently, we studied the effect of the concomitant administration of classic AED (CBZ, PHT and PB) on VPA CL/F. To avoid the complexity of PK interactions caused by multiple-drug coadministration, the influence of a single AED when added to VPA treatment was analyzed. Therefore, the collected data correspond to either mono- or dual therapies.

CBZ, PHT and PB interact with VPA by inducing the metabolism of CYP3A4, CYP2C19, CYP2C9, UGT1A6, UGT1A9 and UGT2B7. This leads to a reduction in plasma VPA levels (CBZ: 39%; PHT and PB: 37%) [5]. Despite the low number of patients undergoing concomitant dual therapy (CBZ = 6.9%; PHT = 2.2% and PB = 2.3% in the development dataset), the effects of the association with CBZ, PHT and PB on the CL/F of VPA were highly determinant of the value of this parameter. In addition, association with LTG was initially included in the model following statistical criteria but was not retained in the final PopPK model due to the lack of clinical relevance, as CL/F of VPA was altered by less than 10%.

In dual therapy with CBZ, a typical VPA CL/F value was estimated at 0.0150 L/h, 0.0157 L/h, 0.818 L/h and 2.258 L/h for typical reference for patients aged 1 year old (mean weight 9.4 kg), 6 years old (mean weight 22.9 kg), 15 years old (mean weight 53.3 kg) and 35 years old (mean weight 73.5 kg), respectively, and 0.010 L/h, 0.103 L/h, 0.538 L/h and 1.484 L/h for the same patients when VPA was administered as monotherapy, resulting in an increase CL/F of about 52%, with the consequent reduction in VPA serum levels. These results are consistent with those calculated using models proposed by other authors, which estimate a 30–43% increase in the CL/F for patients between 5 and 15 years old [7,13,15,16,21,27] and a 40–42% increase for patients over 15 years of age [7,14,18].

In association with PHT, typical VPA CL/F values for the previously mentioned reference patients were estimated at 0.016 L/h, 0.169 L/h, 0.883 L/h and 2.434 L/h, respectively, resulting in an increase in CL/F of about 64%, similar to the 54% reported by Blanco-Serrano 1999 [14], but superior to the 11–43% reported by other authors [9,10,18,24].

In dual therapy with PB, typical VPA CL/F values for the previously mentioned reference patients were estimated at 0.014 L/h, 0.143 L/h, 0.746 L/h and 2.057 L/h, respectively, resulting in an increase in CL/F of about 39%. This is similar to the 24% and 36% reported by several authors [11,14]. However, this increase differs from values (10%, 11%, 12% and 57%) reported by other authors [9,10,16,21].

Considering the significant effect of dual therapy with CBZ, PHT and PB, it would be necessary to reduce the a priori dose of VPA by approximately 52%, 64% or 39% of the standard dose of VPA monotherapy, respectively, to obtain steady-state drug levels within the reference therapeutic range ($C_{min}^{ss}=$ 50–100 mg/L). Despite the significance of these dual-therapeutic effects, no recommendations are given in the reference guidelines for VPA dosing adjustments in patients that take these AEDs in combination [47,48].

According to the literature [5], CLB inhibits the metabolism of VPA, while ESM increases it. However, this effect was not observed in the proposed models, probably due to the low representativeness of these covariates in our population sample, lower than 2% for all age group classifications.

Part of the CL/F IIV seen in the base model (60%) can be explained by the proposed final model. The remaining unexplained PK variability was still relatively large, justifying the need to perform TDM as a strategy to reduce individual PK uncertainty in order to assist in personalized VPA dosage regimens.

The tendency observed in the conditional weighted residuals with respect to population prediction (Figure 1) can be attributed to the fact that many data were taken from the post-monitoring stage, and this biases the residual profile due to the effect of TDM. However, this is not a model misspecification as all other goodness-of-fit measurements appear to indicate the suitability of the model [49].

Before they can be used to reliably optimize dosage, PopPK models must be validated. Both internal and external evaluation procedures were used to successfully assess the suitability of the developed PopPK model for application in dose individualization (Table 3), supporting its implementation in TDM procedures together with Bayesian forecasting according to the correct model performance, adequate precision, accuracy and prediction capability.

The typical population value of VPA CL/F in the monotherapy regimen in the final model was 0.010 L/h, 0.103 L/h, 0.5381 L/h and 1.484 L/h for typical patients of 1 year old (mean weight 9.4 kg), 6 years old (mean weight 22.9 kg), 15 years old (mean weight 53.3 kg) and 35 years old (mean weight 73.5 kg), respectively. These values were aligned with the previous value reported in the scientific literature, where the ranges of CL/F were 0.18–0.37 L/h and 0.22–0.53 L/h in patients younger than 5 years and patients between 6 and 14 years, respectively [1,3,7,10,11,13,15,16,19,21,22,23,27,42,43,44], and 0.38–0.93 L/h in patients ≥ 15 years [8,9,10,13,14,16,17,18,19,21,22,23,24,26,27,42,43]. VPA–protein binding decreases in patients aged over 65, and this increases the free fraction that may be distributed and eliminated, compensating the expected reduction in CL/F in elderly patients due to the inevitable deterioration of liver function with age. The differences found between VPA CL/F in adult and elderly patients were not as significant.

Our findings show the need to adjust the VPA dosage considering the dual therapies with CBZ, PHT or PB to reach therapeutic concentrations ($C_{min}^{ss}\;$≥ 100 mg/L). This fact supports the need for CL/F’s proper characterization in order to optimize VPA dosage regimens.

To the best of our knowledge, this PopPK model of VPA was developed in the largest Caucasian population used to date. The study presented in this manuscript includes a very wide range of ages and a sufficient representation of the most common antiepileptic dual therapies supporting its robustness and wide application in routine clinical practice to improve dosage optimization. Indeed, the VPA PopPK model proposed should be considered for model-informed precision dosing (MIPD) of this drug in Caucasian populations from pediatrics to adults.

Besides all the strengths of the research carried out, some limitations might be acknowledged, such as the retrospective and sparse sampling characteristics of the data, potentially limiting additional evaluations, such as unbound VPA concentrations, additional comedications not taken into account or proper estimation of Ka and V/F. However, the successful internal and external evaluations support the adequate descriptive and predictive capabilities of the final VPA PopPK model developed.

## 5. Conclusions

This study adequately characterized the CL/F of VPA in a large number of Caucasian patients across a very wide range of ages (from 0.1 to 89.4 years old) by using nonlinear mixed-effect modeling and assessing the influence of anthropomorphic, demographic and comedication factors on this parameter. The final PopPK model confirmed the influence of coadministration with carbamazepine, phenytoin and phenobarbital and it demonstrated good stability with an acceptable predictive ability in internal and external evaluations. Based on the final model, which includes development and external evaluation datasets, this PopPK model could be used in the design of a priori VPA dosage regimens and for model-informed precision dosing (MIPD) strategies to optimize VPA treatments in clinical patient management along with Bayesian algorithms during TDM procedures.

## Figures and Tables

**Table 1:** Baseline patients’ characteristics.

Variable	Level	Development	External
Subjects (*n*)		836	368
Age (years)		33.22 ± 23.90; 32.42 (0.11–89.42)	33.48 ± 22.73; 31.58 (0.67–92.92)
Total body weight (kg)		55.82 ± 26.08; 60.00 (6.70–125.00)	58.07 ± 24.59; 62.00 (6.50–110.00)
Height (cm)		149.00 ± 28.58; 160.00 (62.00–194.00)	152.17 ± 26.50; 160.00 (69.00–194.00)
Body mass index * (kg/m^2^)		23.04 ± 5.63; 23.31 (11.81–39.79)	23.36 ± 5.70; 23.36 (11.78–39.61)
Body surface area ** (m^2^)		1.50 ± 0.50; 1.63 (0.36–2.52)	1.55 ± 0.47; 1.68 (0.35–2.39)
VPA daily dose (mg)		1107.13 ± 587.00; 1000.00 (150.00–4500.00)	1177.04 ± 625.21; 1000.00 (200.00–4500.00)
Gender, *n* (%)	Male	451 (53.9)	188 (51.1)
	Female	385 (46.1)	180 (48.9)

* body mass index [29]; ** body surface area [30]; VPA: valproic acid; SD: standard deviation. All continuous covariates are expressed as mean ± SD; median (minimum–maximum).

**Table 2:** PK parameters estimates (development, bootstrap and merged dataset re-estimation).

	Final Model (Development Dataset)			Re-Estimation Final Model (Merged Dataset)			Bootstrap ◊	
Parameter	Estimate	RSE (%)	Shkg (%)	Estimate	RSE (%)	Shkg (%)	Median	95% CI
Ka	Fixed *	-	-	-	-	-	-	-
CL/F	0.646	1.20	-	0.641	1.00	-	0.645	0.631–0.661
AGE	−0.0154	64.3	-	−0.0107	80.2	-	−0.0154	−0.034–0.004
CBZ	0.512	13.4	-	0.549	11.7	-	0.513	0.379–0.658
PB	0.386	23.2	-	0.349	21.1	-	0.398	0.232–0.623
PHT	0.640	24.2	-	0.642	17.8	-	0.638	0.361–0.966
V/F	Fixed **	-	-	-	-	-	-	-
IIV_CL/F (%)	26.8	5.50	19.0	26.4	4.50	19.0	26.6	23.8–29.8
RUV (%)	57.7	3.80	17.0	56.0	3.30	17.0	28.1	25.8–30.4

**◊** Bootstrap *n* = 1000 with successful minimization and no problems for 994 models. * Absorption rate constant (Ka) was fixed at 2.64 h^−1^ for oral solution (syrup), to 0.78 h^−1^ for gastro-resistant tablets and to 0.38 h^−1^ for modified-release coated tablets based on previous information [7,8]. ** Apparent volume of distribution (V/F) was fixed at 14 L for a typical patient of 70 kg [5]. AGE: influence of age on CL/F; CBZ: influence of comedication with carbamazepine (CBZ) on apparent clearance (CL/F) (expressed as a proportion); CI, confidence interval; IIV_CL/F: interindividual variability (IIV) in CL/F (expressed as coefficient of variation in %); PB: influence of comedication with phenobarbital (PB) on CL/F (expressed as a proportion); PHT: influence of comedication with phenytoin (PHT) on CL/F (expressed as a proportion); RSE: relative standard error; RUV: residual unknown variability (expressed as coefficient of variation in %); Shkg: shrinkage. See Equations (1) and (2) for the final CL/F and V/F equations, respectively.

**Table 3:** Summary of precision and bias of the final model in the external evaluation dataset.

AGEC	28 d–2 y		2–11 y		12–18 y		>18 y	
	IPRED	PRED	IPRED	PRED	IPRED	PRED	IPRED	PRED
MPE, %	15.5	34.3	13.8	27.8	15.5	29.2	16.3	33.4
RMSE, %	18.2	37.5	19.6	37.8	19.7	36.4	23.7	56.8

AGEC: age classification group; MPE: median prediction error; RMSE: root mean squared prediction error. These metrics were calculated considering both the individual valproic-acid (VPA)-predicted concentrations (IPRED) and the population-predicted concentrations (PRED).

## Data Availability

Not applicable.

## Supplementary Materials

The following supporting information can be downloaded at: https://www.mdpi.com/article/10.3390/pharmaceutics14040811/s1, Figure S1: Prediction error (PE) boxplot by age group calculated considering both the individual valproic-acid (VPA)-predicted concentrations (IPRED) and the population-predicted concentrations (PRED) using the final PopPK model developed and the external dataset, Table S1: Summary of number of subjects and valproic acid (VPA) concentrations by age group and dataset type, Table S2: Source code of the final model.
