# Supplementary material for: Population Pharmacokinetics of Valproic Acid in Pediatric and Adult Caucasian Patients
Source: Pharmaceutics. 2022 Apr 7;14(4):811. doi: 10.3390/pharmaceutics14040811 (PMC9031051; doi:10.3390/pharmaceutics14040811)
Supplement: Supplementary file 1 [file pharmaceutics-14-00811-s001.zip › pharmaceutics-1649535-supplementary.pdf]

pharmaceutics

MDPI

Supplementary Materials: Population pharmacokinetics of Valproic Acid in pediatric and adult Caucasian patients

Paulo Teixeira-da-Silva, Jonás Samuel Pérez-Blanco, Dolores Santos-Buelga, María José Otero and María José García

Table S1. Summary of number of subjects and valproic acid concentrations by age group and dataset type.

	28d-2y		2-11y		12-18y		>18y		All		
	Dev.	Ext.	Dev.	Ext.	Dev.	Ext.	Dev.	Ext.	Dev.	Ext.	Merg.
Total *	33	11	208	80	70	27	525	250	836	368	1204
	(47)	(12)	(419)	(154)	(157)	(73)	(1128)	(537)	(1751)	(776)	(2527)
Carbamazepine	0	0	3	3	4	2	51	26	58	31	89
	(0)	(0)	(6)	(6)	(6)	(5)	(127)	(53)	(139)	(64)	(203)
Phenytoin	0	0	1	0	1	0	16	11	18	11	29
	(0)	(0)	(1)	(0)	(2)	(0)	(36)	(25)	(39)	(25)	(64)
Phenobarbital	0	0	0	0	1	0	18	9	19	9	28
	(0)	(0)	(0)	(0)	(1)	(0)	(36)	(13)	(37)	(13)	(50)
Lamotrigine	2	0	12	4	6	6	30	12	50	22	72
	(4)	(0)	(34)	(12)	(19)	(16)	(50)	(30)	(107)	(58)	(165)
Topiramate	1	0	2	3	3	2	7	7	13	12	25
	(2)	(0)	(10)	(11)	(9)	(7)	(27)	(18)	(48)	(36)	(84)
Ethosuximide	0	0	2	1	1	1	0	0	3	2	5
	(0)	(0)	(3)	(2)	(2)	(1)	(0)	(0)	(5)	(3)	(8)
Clobazam	1	0	2	0	1	0	4	4	8	4	12
	(2)	(0)	(5)	(0)	(3)	(0)	(6)	(10)	(16)	(10)	(26)
Felbamate	0	0	0	0	0	0	0	0	0	0	0
	(0)	(0)	(0)	(0)	(0)	(0)	(0)	(0)	(0)	(0)	(0)
Primidone	0	0	0	0	0	0	1	0	1	0	1
	(0)	(0)	(0)	(0)	(0)	(0)	(2)	(0)	(2)	(0)	(2)
Other dual	9	6	33	12	7	6	231	98	280	122	402
therapies	(12)	(6)	(78)	(24)	(18)	(23)	(490)	(218)	(598)	(271)	(869)

* Number of patients (number of samples). Dev.: Development dataset; Ext.: External evaluation dataset; Merg.: Merged.

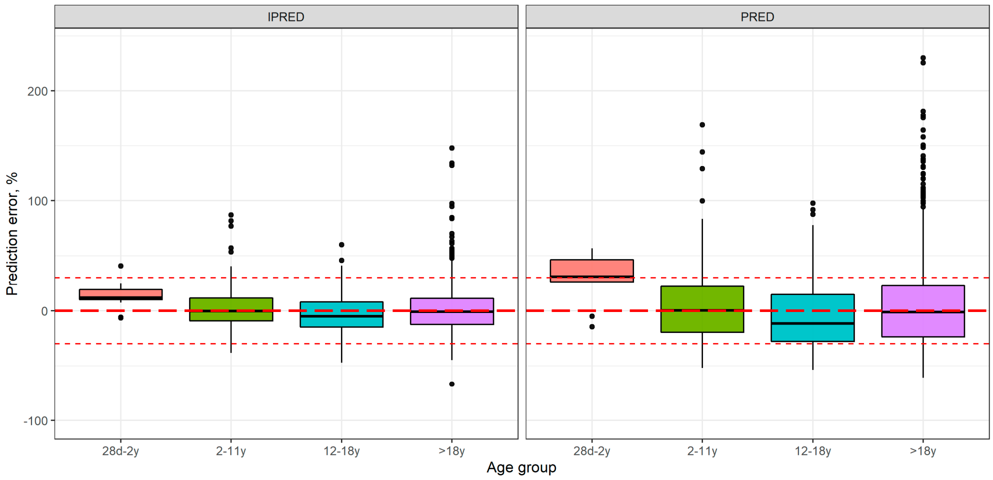

Figure S1. Prediction error (PE) boxplot by age group calculated considering both the individual valproic acid (VPA) predicted concentrations (IPRED) and the population predicted concentrations (PRED) using the final PopPK model developed and the external dataset. Red solid line, PE equal to 0 (unbiased); dashed red lines, PE equal to ± 30% (acceptable bias) 3 outliers (group >18 y) were excluded (PE > 250 %) for better representation. ●*28d-2y* ●*2-11y* ●*12-18y* ●*>18y*.

Table S2. Source code of the final model.

$PROBLEM RUN00X - FINAL MODEL
;; 1. Description: RUN00X - Final model

```
$INPUT
C,ID,DAT1=DROP,TIME,AMT,II,EVID,MDV,ADDL,SS,DV=DROP,OCC,AGE,SEX,TBW,HGT,BMI,
BSA,IBW,CBZ,PHT,PB,ESM,LTG,TPM,CLB,FBM,PRM,OMC,DCBZ,DPHT,DPB,RPO,DDV,FFS,LN
DV=DV,TSFD,TSLD,RATE=DROP
$DATA D:\PKPD\VPA\data\NONMEM\VPA_ALL.csv
IGNORE=C
$SUBROUTINE ADVAN2 TRANS2
$PK
;;; CLPHT-DEFINITION START
IF(PHT.EQ.0) CLPHT = 1
IF(PHT.EQ.1) CLPHT = ( 1 + THETA(6))
;;; CLPHT-DEFINITION END
;;; CLPB-DEFINITION START
IF(PB.EQ.0) CLPB = 1
IF(PB.EQ.1) CLPB = ( 1 + THETA(5))
;;; CLPB-DEFINITION END
;;; CLCBZ-DEFINITION START
IF(CBZ.EQ.0) CLCBZ = 1
IF(CBZ.EQ.1) CLCBZ = ( 1 + THETA(4))
;;; CLCBZ-DEFINITION END
;;; CLAGE-DEFINITION START
   CLAGE = ((AGE/15)**THETA(3))
;;; CLAGE-DEFINITION END
;;; CL-RELATION START
CLCOV=CLAGE*CLCBZ*CLPB*CLPHT
```

```
;;; CL-RELATION END
TVCL = THETA(1) * (TBW/70)**0.75
TVCL = CLCOV*TVCL
TVV   = THETA(2) * (TBW/70)**1
CL     = TVCL * EXP(ETA(1))
V       = TVV   * EXP(ETA(2))
IF (FFS.EQ.0) KA=0.78 ;N/A [gastro-resistant tablets]
IF (FFS.EQ.1) KA=2.64 ;oral solution (syrup) (Fixed - Ding J 2015)
IF (FFS.EQ.2) KA=0.78 ;gastro-resistant tablets (Fixed - Methaneethorn J 2017)
IF (FFS.EQ.3) KA=0.38 ;modified release coated tablets (Fixed - Methaneethorn J 2017)
K=CL/V
SC=V
$ERROR
IPRED = -5                              ; arbitrary value; to prevent from run stop due to log
IF (F.GT.0) IPRED = LOG(F)
Y = IPRED + EPS(1)
$THETA   (0,0.650064) ; CL
  14 FIX ; V
$THETA   (-100,-0.0456768,100000) ; CLAGE1
$THETA   (-1,0.549962,5) ; CLCBZ1
$THETA   (-1,0.438353,5) ; CLPB1
$THETA   (-1,0.528116,5) ; CLPHT1
$OMEGA   0.0535612
  0   FIX
$SIGMA   0.0631143
$ESTIMATION METHOD=1 INTERACTION NOABORT MAXEVAL=9999 SIGDIG=3 PRINT=5
POSTHOC SADDLE_RESET=1 ; INTERACTION NOT NEEDED IN LTBS
$COVARIANCE
$TABLE ID TIME TSLD MDV DV PRED WRES IPRED CWRES CL V ETA1 ETA2 AGE TBW HGT
BMI BSA IBW DCBZ DPHT DPB DDV
SEX CBZ PHT PB ESM LTG TPM CLB FBM PRM OMC RPO FFS NOPRINT ONEHEADER NOAP-
PEND FORMAT=, FILE=SDTABX.csv
```

SEX: gender; TBW: total body weight; HGT: height; BMI: body mass index; BSA: body surface area; IBW: ideal body weight; CBZ: carbamazepine; PHT: phenytoin; PB: phenobarbital; ESM: ethosuximide; LTG: lamotrigine; TPM: topiramate; CLB: clobazam; FBM: felbamate; PRM: primidone; OMC: other dual therapies; DCBZ: daily dose of carbamazepine; DPHT: daily dose of phenytoin; DPB: daily dose of phenobarbital; RPO: dosage regimen; DDV: daily dose of valproic acid; FFS: pharmaceutical forms; DV: concentration.
